# Investigation of Cytotoxicity, Oxidative Stress, and Inflammatory Responses of Tantalum Nanoparticles in THP-1-Derived Macrophages

**DOI:** 10.1155/2020/3824593

**Published:** 2020-12-03

**Authors:** Li Zhang, El-Mustapha Haddouti, Hannes Beckert, Ralf Biehl, Shyam Pariyar, Julian M. Rüwald, Xian Li, Max Jaenisch, Christof Burger, Dieter C. Wirtz, Koroush Kabir, Frank A. Schildberg

**Affiliations:** ^1^Clinic for Orthopedics and Trauma Surgery, University Hospital Bonn, 53127 Bonn, Germany; ^2^Microscopy Core Facility, Medical Faculty, Bonn Technology Campus Life Sciences, University of Bonn, 53127 Bonn, Germany; ^3^Jülich Centre for Neutron Science (JCNS-1) & Institute of Biological Information Processing (IBI-8), Forschungszentrum Jülich, 52425 Jülich, Germany; ^4^Department of Horticultural Sciences, University of Bonn, 53121 Bonn, Germany; ^5^Department of Orthopedic and Trauma Surgery, Xiyuan Hospital, China Academy of Chinese Medical Science, 100091 Beijing, China

## Abstract

Tantalum (Ta) is gaining attention as a biomaterial in bone tissue engineering. Although the clinical advantage of Ta-based implants for primary and revision total joint replacement (TJA) has been well documented, few studies investigated the effect of wear products of Ta implants on peri-implant cells, and their potential contribution to aseptic implant loosening. This study is aimed at examining the cytotoxicity, oxidative stress, and proinflammatory potential of Ta and TiO_2_ nanoparticles (NPs) on macrophages *in vitro*. NPs were characterized using scanning electron microscopy, dynamic light scattering, and energy-dispersive X-ray. To test the NP-mediated cellular response in macrophages, THP-1-derived macrophages were challenged with both NPs, and cytotoxicity was analyzed using CCK-8 and LDH assays. Flow cytometry was used to investigate particle uptake and their internalization routes. NP-mediated oxidative stress was investigated by measuring the production of reactive oxygen species, and their proinflammatory potential was determined by quantifying the production of TNF*α* and IL-1*β* in cell culture supernatants using ELISA. We found that both Ta and TiO_2_ NPs were taken up through actin-dependent phagocytosis, although TiO_2_ NPs did also show some involvement of macropinocytosis and clathrin-mediated endocytosis. Ta NPs caused no apparent toxicity, while TiO_2_ NPs demonstrated significant cytotoxicity at a concentration of over 100*μ*g/mL at 24 h. Ta NPs induced negligible ROS generation and proinflammatory cytokines (TNF*α*, IL-1*β*) in macrophages. In contrast, TiO_2_ NPs markedly induced these effects in a dose-dependent manner. Our findings indicate that Ta NPs are inert, nontoxic, and noninflammatory. Therefore, Ta could be considered an excellent biomaterial in primary and revision joint arthroplasty implants.

## 1. Introduction

Aseptic loosening is the leading cause of revision surgery and plays a predominant role in limiting the longevity of current total joint arthroplasty (TJA). Wear particles have been recognized as one of the major factors responsible for aseptic implant loosening [1]. After implantation, orthopedic prosthesis becomes an internal source of wear particles [2–5]. Upon corrosion and abrasion, nondegradable biomaterial wear particles are inevitably released in adjacent peri-implant tissues or systemically disseminated, inducing local and systemic reactions [6–8]. They represent a long-term hazard that interacts with peri-implant cell lineages such as macrophages, fibroblasts, osteoblasts, osteoclasts, and mesenchymal stem cells (MSCs). This process may disrupt local cellular functions; create chronic inflammation, which favors periprosthetic osteolysis; and eventually leads to aseptic implant loosening with subsequent revision surgery. Clinically, as the only established treatment for periprosthetic aseptic loosening, revision TJA is technically demanding and associated with high complication rates, high morbidity, and poor clinical and functional outcomes. Moreover, because of the complexity of the procedure, compromised soft tissue, and bone defects, revision TJA has a greater failure rate than primary TJA [9]. Therefore, choosing the appropriate implant biomaterial is critical for the long-term survival of both primary and revision TJAs.

Tantalum (Ta) is described as an “extremely bioinert” material and has been widely applied as artificial joints, endovascular stents, and coating [10–12]. As biomaterial for implant components in primary and revision TJA, Ta can be formed with a highly porous structure that could mimic the structure of cancellous bone. Similar to titanium (Ti), Ta provides outstanding biocompatibility and corrosion resistance [13, 14]. Moreover, porous Ta components offer lower elastic modulus and higher surface frictional characteristics than conventional Ti implants, thus reducing shielding and improving early stability. These properties make Ta an ideal choice in TJA revision surgery [15, 16]. Recent studies on failed Ta implant revision hip arthroplasty described nanoscale Ta fragments (diameters ranging from 9.6 to 243.5 nm) released from the implant surface [17, 18]. Because of this phagocytozable size range and spatial proximity, Ta nanoparticles (NPs) could be internalized by peri-implant cells, e.g., macrophages, fibroblasts, and MSCs, and provoke hazardous cellular responses. However, the difficulties in purifying and characterizing NPs until today result in an underestimation of the adverse impact of Ta NPs. Therefore, particular attention must be paid to nanoscale Ta wear particles' potentially hazardous effect on peri-implant cells and their potential contribution to repeated prosthetic loosening and subsequent rerevision [19]. However, to date, this topic remains largely unknown.

Macrophages are the critical cells associated with wear particle-induced aseptic loosening. As sentinels of the innate immune system, macrophages are the first immune cells involved in aseptic loosening by recognizing, internalizing, and getting activated upon wear particle exposure [20, 21]. Once activated, macrophages exert an increased proinflammatory phenotype and initiate a chronic inflammatory response characterized by the release of proinflammatory mediators, such as TNF*α*, IL-1*β*, monocyte chemotactic protein-1 (MCP-1), and IL-8 [21, 22]. These reactions create an inflammatory microenvironment that facilitates elevated osteoclastic bone destruction, suppressed bone formation, and ultimately lead to aseptic implant loosening. Therefore, an attempt to elucidate macrophages' response to biomaterial wear debris is critical to understanding the pathology of implant loosening.

The biological response of peri-implant cells to implants is critical for early and late implant success. Recently, emerging *in vitro* and *in vivo* studies on osteoblasts and MSCs have demonstrated the Ta-based implants' advantages over commonly used Ti-based implants [23–25]. However, limited research investigated the interaction between Ta implants and other peri-implant cells, such as macrophages. In particular, the biological response of macrophages to Ta implants' wear products, such as nanoscale Ta particles and ions, has never been elucidated. Therefore, this study is aimed at analyzing the effects of Ta NPs on macrophage biology using the THP-1 cell line, an *in vitro* cell model that is well known, reproducible, and readily available to different labs. To this end, we investigated Ta NPs' uptake routes, cytotoxicity, oxidative stress, and proinflammatory potential on THP-1-derived macrophages *in vitro*.

## 2. Materials and Methods

### 2.1. Cell Culture and Differentiation

THP-1 cells (American Type Culture Collection, Manassas, VA, USA), a human monocytic leukemia-derived cell line, were cultured in RPMI 1640 medium supplemented with 10% fetal bovine serum and 1% penicillin/streptomycin at 37°C in a humidified atmosphere with 5% CO_2_. For macrophage differentiation, the THP-1 cells were stimulated with 50ng/mL phorbol 12-myristate 13-acetate (PMA) (Sigma-Aldrich, Taufkirchen, Germany) overnight.

### 2.2. Particle Preparation

Ta (NM-0036-HP) and TiO_2_ (NO-0046-HP) nanosized particles were obtained from IoLiTec (Heilbronn, Germany), weighed into autoclaved tubes, and then sterilized by radiation. Stock dispersions (50 mg/mL) were prepared in phosphate-buffered saline (PBS) solution, followed by 20min of continuous sonication using the Emmi-12HC (EMAG AG, Germany) bath sonicator operating at 45kHz at 30°C. Then, the stock solution was stored at 4°C in the dark. Final dispersions were prepared from a serial dilution of the stock in full cell culture medium followed by another 20min sonication at 30°C and vigorous vortexing for 5s immediately before adding them to the cells.

### 2.3. Characterization of Particles

The physicochemical properties of particles were analyzed with scanning electron microscopy (SEM) and dynamic light scattering (DLS). The particle morphology and size distribution were determined with SEM. Samples dispersed in ddH_2_O were vortexed before applying 2*μ*L of the mixture onto a silicon wafer. After drying the sample, the wafer was sputtered with an ~2nm platinum layer in a Leica ACE600 sputter coater in an argon atmosphere to prepare it for high-resolution field-emission scanning electron microscopy (FESEM) (Crossbeam 550, Zeiss). A Schottky emitter-based field emission Gemini II electron column (Zeiss) was used with 0.8kV acceleration voltages and currents between 150pA and 250pA for imaging. The samples were imaged with the InLens SE (secondary electrons) detector (Zeiss) for topographic imaging (working distance < 3 mm). ImageJ (NIH) software was used to determine the size distribution of NPs by randomly selecting 100 particles from the SEM images. The size distribution of NPs was fitted using a Gaussian distribution in GraphPad Prism 7 software (GraphPad, La Jolla, CA, USA).

The DLS experiment was carried out with Zetasizer Nano-ZS (Malvern, Worcestershire, Great Britain), which estimates the size distribution from the measured correlation function by nonnegative least square (NNLS) analysis. TiO_2_ and Ta NPs were dispersed in ddH_2_O, PBS, RPMI 1640, and RPMI 1640 containing 10% FBS. Then, samples of NPs were mixed thoroughly via sonication and vortexing before measuring them at 250*μ*g/mL. The scattered light is monitored at an angle of 173° with a wavelength of 633 nm. Measurements were done at 20°C at an average of 10-20 short frames of 10 s. The observed decay rate is Γ = *q*^2^*D*_0_ with scattering vector *q*. The hydrodynamic radius *R*_*h*_ is related to the diffusion coefficient *D*_0_ according to the Stokes-Einstein equation *D*_0_ = *k*_B_*T*/6*πηR*_*h*_ with the Boltzmann constant *k*_B_, temperature *T* (in K), and viscosity *η* of the solvent.

The energy-dispersive X-ray (EDX) analysis was performed using the EDX system (EDAX, Ametek GmbH, Meerbusch, Germany). The EDX system is fitted with a Super Ultrathin Window Si-(Li) detector with a resolution < 138 eV (MnK*α* at 1000 cps), configured with a take-off angle of 45° relative to the microscope stage. The Genesis 4000 software (version 3.61) was used to display and evaluate the collected spectra. Measuring adjustments (dwell time = 100 *μ*s; amplifier integration time = 100 *μ*s; reads = 100; and map resolution = 512 × 256 pixels) remained constant during the measurements. The time required for the analysis of each sample was 655 live seconds. Al, Ta, Si, Ti, Fe, Co, and Ni, were chosen for quantification, whereas the other elements such as carbon, oxygen, nitrogen, potassium, and magnesium were not considered (Supplementary Figure 2). Quantification of the elements was standardless using the ZAF-algorithm and selecting the automatic integration of the obtained spectra. The evaluated parameters were the standardized amounts of Ta and Ti.

### 2.4. Endotoxin Test

Endotoxin levels in nanoparticle samples were quantified with the Limulus amebocyte lysate (LAL) assay using the ToxinSensor™ Chromogenic LAL Endotoxin Assay Kit (Genscript) according to the manufacturer's instructions with modifications. This kit has a minimum endotoxin detection limit of 0.01EU/mL and a measurable concentration range of 0.01 to 1EU/mL. 100*μ*g raw NPs were suspended in 1mL endotoxin-free water at 100*μ*g/mL. A volume of 100*μ*L NP suspension and 100*μ*L of endotoxin standard samples derived from *Escherichia coli* (0.01-0.1 unit/mL) were incubated with 100*μ*L reconstituted LAL reagent for 30min at 37°C and a volume of 100*μ*L endotoxin-free water as control. After the initial incubation, 100*μ*L reconstituted chromogenic substrate solution was added into each vial, and the incubation continued for an additional 6min. The reaction was stopped by adding 500*μ*L of reconstituted color-stabilizer #1 (stop solution). Then, 500*μ*L of reconstituted color-stabilizer #2 and #3 were added. Importantly, particles were removed by two rounds of centrifugation (2500 rpm for 15 min) as they may interfere with the absorbance value [26]. Finally, the absorbance value of each reaction was determined at 545nm using a microplate reader. Distilled water was used as a blank to adjust the photometer to zero absorbance. All samples (100*μ*L) were analyzed in duplicate. Only tests producing a correlation coefficient for the standard curve of 0.98 or greater were accepted. Because NPs may interfere with the endotoxin measurement, all NP samples were measured with and without aliquots of a test sample containing a known amount of the endotoxin (0.005 EU/mL). The assays were considered reliable if the recovery of spikes was 80-120%.

### 2.5. Cell Viability Assay

The CCK-8 Cell Counting Kit (Dojindo, Japan) was used to evaluate the viability of macrophages that were treated with TiO_2_ and Ta NPs. Briefly, THP-1 cells were seeded in 96-well cell culture plates (0.8 × 10^5^ cells/well) with PMA and incubated overnight for differentiation. Cells were then challenged with a series of concentrations of NPs (20, 50, 100, 200, and 500*μ*g/mL and 0.2mL/well) for 1h, h, 6h, 1d, 3d, and 7d. At the end of each time point, the medium was replaced with fresh culture medium containing CCK-8 solution (1 : 10 in culture medium), and further incubated for another hour at 37°C and 5% CO_2_. The supernatant was collected and transferred to another 96-well plate to avoid the interference of NPs on optical density (OD) reading. Finally, absorbance was measured at 450 nm using a microplate reader (Tecan, Männedorf, Switzerland). The viability of the nonchallenged cells was considered 100%.

### 2.6. Lactate Dehydrogenase Release Assay

Cell culture supernatants from THP-1-derived macrophages were collected after 1, 3, and 7 days after NP exposure. Resulting supernatants were evaluated for LDH activity using the Cytotoxicity LDH Assay Kit-WST (Dojindo, Japan). The absorbance of all samples at a wavelength of 490 nm was recorded using a microplate reader. Low controls (untreated cells) and high controls (cells treated with lysis buffer) were used to calculate the cell mortality:

(1) $$\begin{array}{c} \text{Cytotoxicity }\left(\%\right)=\frac{\text{test substance}-\text{low control}}{\text{high control}-\text{low control}}\times 100. \end{array}$$

### 2.7. Measurement of Nanoparticle Uptake by Flow Cytometry

Particle uptake by macrophages was measured via flow cytometry. THP-1 cells were seeded into 24-well cell culture plates at 2 × 10^5^ cells per well with PMA overnight. Then, THP-1 cells were preincubated for 30min with the following uptake inhibitors: 25 *μ*M cytochalasin D (CytD), used to disrupt actin-dependent phagocytosis [27]; 100 *μ*M amiloride (Ame), applied as an inhibitor of micropinocytosis [28]; and 25*μ*M genistein (Gen) and 25 *μ*M chlorpromazine hydrochloride (Cpz), used to inhibit caveolae- and clathrin-mediated endocytosis, respectively [29]. Cells were subsequently exposed to TiO_2_ or Ta NPs at concentrations of 100 and 500*μ*g/mL (0.5mL/well) for 1 and 6h. The inhibitors were not removed during the uptake experiments. At the end of each time point, cells were trypsinized and centrifuged. The resulting cell pellet was resuspended in 200*μ*L ice-cold PBS and analyzed with flow cytometry. Flow cytometry data were acquired using FACSCanto II using FACS-Diva software and analyzed using FlowJo software (all from BD Biosciences). Cell profiles were investigated through forward scatter (FSC) vs. side scatter (SSC) to exclude cell debris and free particles. Mean SSC was used as a measure of particle uptake. The increase in SSC, which was directly related to the cellular granularity, was analyzed as described previously [30]. SSC increases at 4°C, which indicates the passive, energy-independent entrance of particles into cells, and a portion of particles adherent to macrophages' outer membranes were analyzed and subtracted from the data acquired at 37°C.

### 2.8. Bright-Field Microscopy

THP-1 cells were differentiated as described above in a 12-well chamber slide (35mm) (ibidi, Germany) and treated for 24 h with standard culture medium, TiO_2_, and Ta NPs. Bright-field microscope images were taken using an IX81 microscope (Olympus).

### 2.9. Measurement of Intracellular ROS Generation

The generation of intracellular ROS was measured using 2′,7′-dichlorodihydrofluorescein diacetate (DCFH-DA, Sigma-Aldrich). PMA-differentiated THP-1 cells (0.8 × 10^5^ cells/well in 96-well plates) were washed with warm PBS and then treated with TiO_2_ or Ta NPs at 50 to 500*μ*g/mL for 1, 3, 6, and 24h. N-Acetyl-L-cysteine (NAC) was applied in some experiments to inhibit ROS production. For these tests, PMA-differentiated THP-1 cells were pretreated with 10 mM NAC for 30min and then stimulated with 100 or 500*μ*g/mL NPs for another 6 or 24h in the presence of 10 mM NAC. Then, serum-free RPMI medium containing 20*μ*M DCFH-DA was added to the samples and controls for another 30min under standard culturing conditions. Untreated controls were maintained for each time interval. Subsequently, cells were carefully washed twice with prewarmed PBS. The fluorescence intensity of the resulting fluorescent product dichlorofluorescein (DHF) was measured with a microplate reader at the excitation and emission wavelengths of 485 and 530 nm, respectively.

### 2.10. Enzyme-Linked Immunosorbent Assay (ELISA)

PMA-differentiated THP-1 cells (0.8 × 10^5^ cells/well of a 96-well plate) were firstly primed with 200ng/mL LPS (LPS-EK Ultrapure, InvivoGen) or incubated with standard culture medium for 3 h. Then, LPS was removed, and macrophages were treated with particles for 1h, 3h, 6h, 1d, 3d, and 7d. NAC was applied in some experiments to inhibit ROS in THP-1 macrophages. For these tests, PMA-differentiated THP-1 cells were pretreated with 10mM NAC for 30min following LPS priming and then stimulated with 100 or 500*μ*g/mL NPs for another 6 h in the presence of 10mM NAC. Cell-free supernatants were collected and centrifuged (200 × g, 10min, 4°C), and aliquots were stored at -80°C. TNF*α* and IL-1*β* were determined with an ELISA kit (R&D Systems, Wiesbaden, Germany) according to the manufacturer's protocol, using a microplate ELISA reader.

### 2.11. Statistical Analysis

GraphPad Prism 7 software was used for statistical analysis. All values were expressed as the mean + SD. Student's *t*-test was used for comparisons between two groups, and one-way ANOVA was used to determine statistical differences between several groups. Differences were considered significant at ^∗^*p* < 0.05, ^∗∗^*p* < 0.01, and ^∗∗∗^*p* < 0.001.

## 3. Results

### 3.1. Nanoparticle Characterization

**Figure 1:**
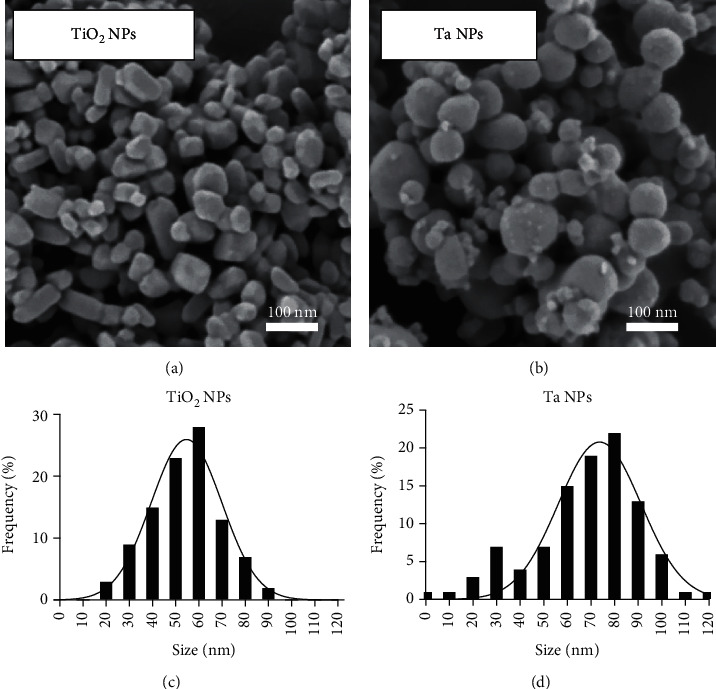
Characterization of TiO_2_ and Ta NPs. (a, b) Scanning electron microscopic (SEM) images of TiO_2_ NPs and Ta NPs. (c, d) Particle size distribution with Gaussian fitting of TiO_2_ NPs and Ta NPs was determined by randomly selecting 100 particles from the SEM images.

SEM images of TiO_2_ NPs (Figure 1(a)) and Ta NPs (Figure 1(b)) exhibited a spherical shape. TiO_2_ NPs were slightly more plate-like, with less rounded features. The average size of the TiO_2_ and Ta particles estimated from SEM images was 54.3 ± 14.6 nm (Figure 1(c)) and 67.9 ± 22.1 nm (Figure 1(d)), respectively. To further characterize the size distribution of particles in the liquid phase, TiO_2_ and Ta NPs were analyzed using dynamic light scattering (DLS). The hydrodynamic sizes of TiO_2_ and Ta NPs in ddH_2_O, PBS, RPMI 1640 growth medium, and RPMI 1640 growth medium containing 10% FBS are summarized in Supplementary Table 1. The DLS results demonstrated that both NPs had the tendency to form aggregates in different solutions. In addition, endotoxin levels were also tested in both groups by employing the LAL chromogenic assay. All NP samples did not show any contamination with endotoxins (Supplementary Figure 1), confirming that all particle samples could be considered endotoxin-free. To confirm NP purity, energy-dispersive X-ray (EDX) analyses were performed. The resulting EDX spectra of NPs indicated the presence of Ti and Ta as the main elements, with no indication of other selected elements (Supplementary Figure 2). Notably, our EDX data revealed some extent of oxidation of Ta NPs, which may be attributed to the formation of an oxide layer on the surface of Ta NPs during sample preparation.

### 3.2. Bright-Field Microscopy of THP-1-Derived Macrophages Exposed to TiO_2_ and Ta NPs

**Figure 2:**
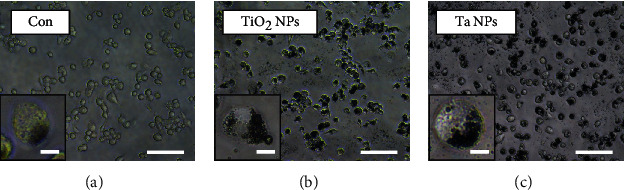
Bright-field microscope images of THP-1-derived macrophages treated with TiO_2_ and Ta NPs. THP-1-derived macrophages were cultured in the presence of (a) standard culture medium (Con: control), (b) TiO_2_ NPs, and (c) Ta NPs for 24 h. Bright-field images were taken 24h after exposure. Scale bar: 100*μ*m (overview) and 10*μ*m (enlarged insert).

To observe the interaction between THP-1-derived macrophages and NPs, THP-1-derived macrophages were exposed to culture medium (Figure 2(a)), TiO_2_ NPs (Figure 2(b)), and Ta NPs (Figure 2(c)) for 24h, and bright-field images were taken. Bright-field images showed the presence of TiO_2_ and Ta NP agglomerates in the presence of THP-1-derived macrophages.

### 3.3. Analysis of the Cytotoxic Effect of TiO_2_ and Ta NPs in THP-1-Derived Macrophages

**Figure 3:**
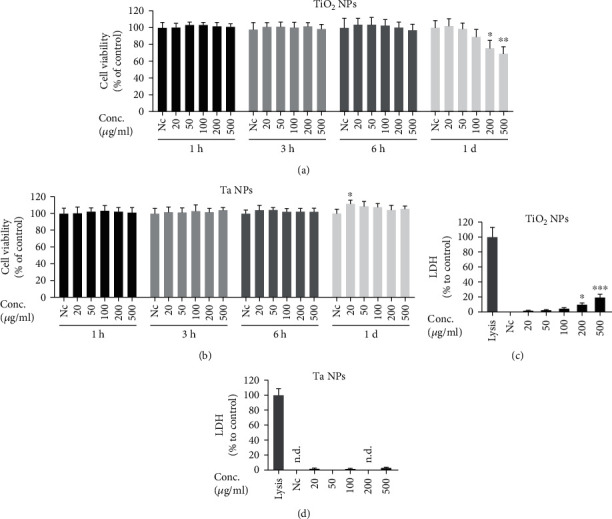
Analysis of cytotoxic effects of TiO_2_ and Ta NPs on macrophages. (a, b) Cell viability was determined by CCK-8 assay at indicated time points. (c, d) Lactate dehydrogenase (LDH) leakage was evaluated by LDH assay after 24h. Viability and LDH release are normalized and expressed as mean + SD as percentage of untreated cells of three independent experiments (^∗^*p* < 0.05, ^∗∗^*p* < 0.01, and ^∗∗∗^*p* < 0.001). n.d.: not detectable. Nc: nontreated control.

THP-1-derived macrophages were exposed to TiO_2_ and Ta NPs using a concentration range of 20-500*μ*g/mL. Cell viability was evaluated by CCK-8 assay. TiO_2_ NPs demonstrated no dose-dependent cytotoxicity until they reached a threshold of 200*μ*g/mL at 24 hours (Figure 3(a)). Beyond this limit, TiO_2_ NPs significantly decreased macrophage viability in a dose- and time-dependent manner compared to untreated cells (Supplementary Figure 3(a)). In contrast, exposure of macrophages to 20-500 *μ*g/mL Ta NPs did not result in a significant decrease in cell viability (Figure 3(b), Supplementary Figure 3(b)) until 7 days. Notably, at 24 and 48h using 20*μ*g/mL, cell viability was even slightly increased (Supplementary Figure 3(b)). As Ta's density is higher than that of TiO_2_, we also compared 100*μ*g/mL TiO_2_ with 500*μ*g/mL Ta to compensate a potential bias by NP density. Similar to the direct comparison between the same NP concentrations, cells were still less viable in the presence of 100*μ*g/mL TiO_2_ than in the presence of 500*μ*g/mL Ta NPs.

LDH is being released by cells as a consequence of cell membrane damage, and therefore, it is an indicator of irreversible cell death. As expected, the LDH release assay congruently supported our CCK-8 cell viability results. Compared to untreated cells, in the TiO_2_ NP group, there was no appreciable cell death until 24 hours. Then, increasing cell death was observed in a dose- and time-dependent manner (Figure 3(c), Supplementary Figure 3(c)). In contrast, exposure of macrophages to 20-500*μ*g/mL Ta NPs for up to 7 days did not result in a significant increase in cell death (Figure 3(d), Supplementary Figure 3(d)). Overall, TiO_2_ and Ta NPs demonstrated good biocompatibility at low concentrations. At high concentrations, however, only TiO_2_ NPs led to cytotoxicity.

### 3.4. Cellular Uptake and Internalization Routes of TiO_2_ and Ta NPs

**Figure 4:**
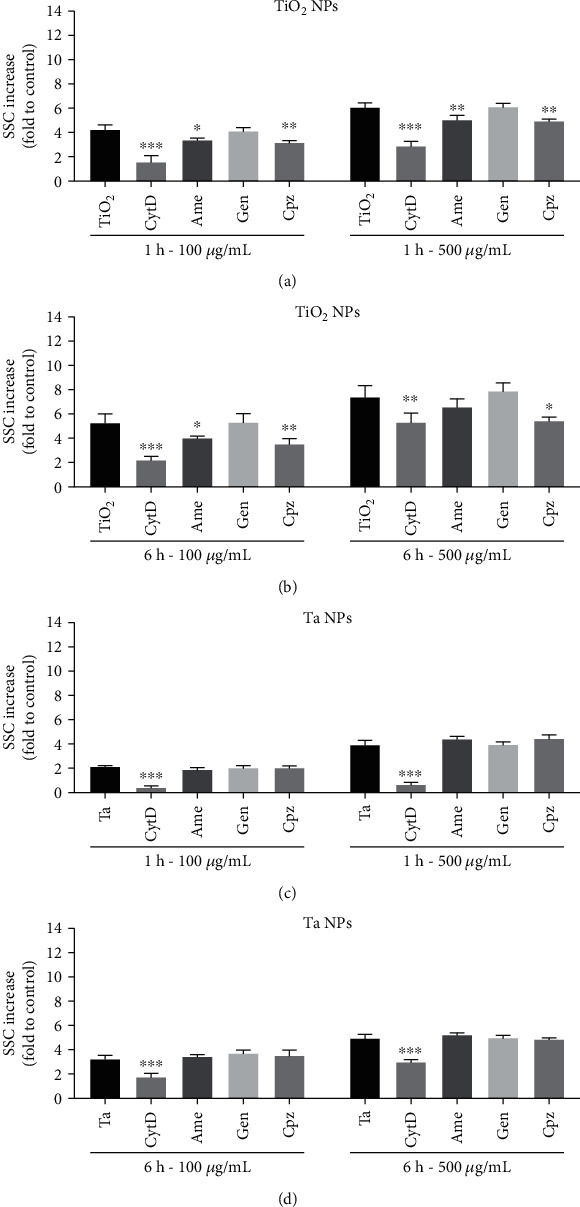
TiO_2_ and Ta NP internalization and their particle-specific uptake routes. THP-1-derived macrophages were pretreated with different uptake inhibitors for 30min and then exposed to (a) TiO_2_ and (b) Ta NPs at 100 and 500*μ*g/mL for 1 and 6 h. Changes in side scatter (SSC) were analyzed with flow cytometry. SSC changes at 4°C were subtracted, and data were expressed as mean + SD as fold of NP-untreated cells (Nc) of three independent experiments. Statistical differences were shown as ^∗^*p* < 0.05, ^∗∗^*p* < 0.01, and ^∗∗∗^*p* < 0.001 for inhibitor-treated groups vs. cells treated with particles only.

Macrophages are phagocytic cells capable of sensing and internalizing particulate matters. To validate the uptake of TiO_2_ and Ta NPs, THP-1 macrophages were exposed to TiO_2_ and Ta NPs (100 and 500 *μ*g/mL) for 1 and 6 h, and flow cytometry was used to quantify NP uptake. Flow cytometric analysis demonstrated that both TiO_2_ and Ta NPs significantly enhanced cell granularity, which was detected by SSC signals and can be used as a readout for NP uptake. We observed significantly lower SSC signals in the Ta NP group in contrast to the TiO_2_ group (Figure 4) at 1 and 6 h, suggesting that THP-1-derived macrophages took up more TiO_2_ NPs than Ta NPs at the same concentration.

To determine possible uptake routes of TiO_2_ and Ta NPs, THP-1-derived macrophages were preincubated with different uptake inhibitors before NP exposure. Comparison of the FACS analysis data at 1 and 6 h demonstrated that the majority of the particles were internalized within the first hour (Figure 4). To ensure that the uptake inhibitors did not affect cell viability, cell viability was tested in the presence of different inhibitor concentrations up to 6 hours (Supplementary Figure 4).

As shown in Figures 4(a) and 4(b), the uptake of TiO_2_ and Ta NPs was significantly reduced after preincubation with CytD, Ame, and Cpz, but not Gen, suggesting that macrophages take up TiO_2_ NPs via phagocytosis, macropinocytosis, and clathrin-dependent endocytosis. Similarly, Ta NPs were internalized through phagocytosis (Figures 4(c) and 4(d)). However, macropinocytosis and clathrin- and caveolin-mediated endocytosis inhibitors did not significantly reduce Ta uptake, indicating the absence of these mechanisms. In summary, the active internalization of TiO_2_ and Ta NPs were dominated by phagocytosis. TiO_2_ NP uptake also involved macropinocytosis as well as clathrin-mediated endocytosis, but to less extent. Caveolae-mediated endocytosis seems not to be involved in the uptake of both particles.

### 3.5. Ta NPs Trigger Less ROS Production than TiO_2_ NPs

**Figure 5:**
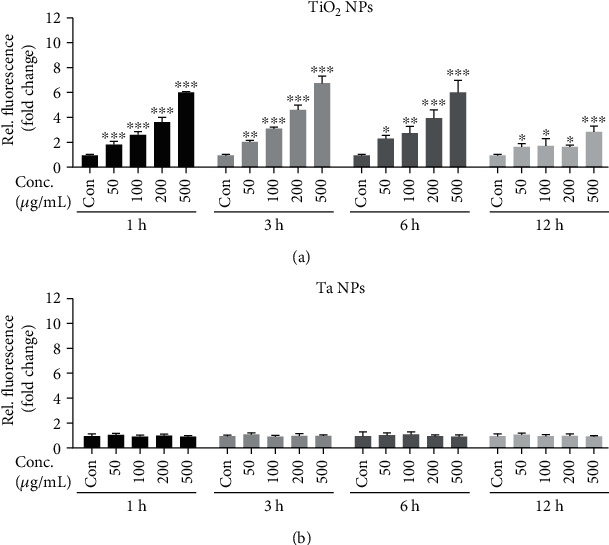
Endogenous ROS generation in THP-1-derived macrophages after TiO_2_ and Ta NP exposure. THP-1-derived macrophages were stimulated with (a) TiO_2_ and (b) Ta NPs (50-500*μ*g/mL) for 1, 3, 6, and 24h. Cells were stained with DCFH-DA (20*μ*M) for 30min, and then fluorescence intensity was analyzed with a microplate reader. Fluorescence values are normalized and expressed as mean + SD as fold of untreated cells of three independent experiments (^∗^*p* < 0.05, ^∗∗^*p* < 0.01, and ^∗∗∗^*p* < 0.001).

NP-induced oxidative stress contributes to nanopathology [31]. In this study, the overall intracellular ROS elevation after NP challenge was examined using the probe 2′,7′-dichlorodihydrofluorescein diacetate (DCFH-DA). In our setup, as shown in Figure 5(a), TiO_2_ NPs dose-dependently induced robust intracellular ROS generation at different time points. Comparison of these data showed that the majority of ROS was produced within the first hour after NP treatment. Notably, a reduction of endogenous ROS production was observed at 24 hours. This may result due to a decrease in cell viability (Figure 3(a)) at this time point. In contrast, Ta NPs induced negligible intracellular ROS elevation at different concentrations and incubation periods (Figure 5(b)). These results indicated that, compared to TiO_2_ NPs, Ta NPs are “inert” in generating ROS.

### 3.6. Ta NPs Exert Less Proinflammatory Activity than TiO_2_ NPs *In Vitro*

The wear particle-induced inflammatory response, mainly driven by macrophages, underlies the pathology of periprosthetic osteolysis and aseptic loosening [32]. Among all the proinflammatory mediators, TNF*α* and IL-1*β* are the primary initiators and significant mediators of the wear particle-induced inflammatory cascade. Therefore, we investigated the proinflammatory effect of TiO_2_ and Ta NPs by evaluating their induction of TNF*α* and IL-1*β* with or without LPS priming.

**Figure 6:**
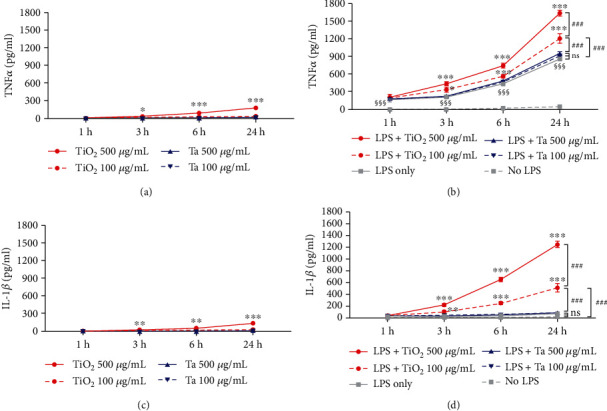
Proinflammatory potential of TiO_2_ and Ta NPs on THP-1-derived macrophages. THP-1-derived macrophages were primed with 200 ng/mL LPS for 3 h, washed to remove the LPS, and then incubated with TiO_2_ or Ta NPs using the indicated doses for up to 24 h. Release of (a, b) TNF*α* and (c, d) IL-1*β* were measured by ELISA. THP-1-derived macrophages without LPS stimulation were used as controls. ^∗^ indicates significant differences of different time points of the TiO_2_ NP group compared to the TiO_2_ NP 1 h group (^∗^*p* < 0.05, ^∗∗^*p* < 0.01, and ^∗∗∗^*p* < 0.001). # indicates significant differences compared to the TiO_2_ NP (100 *μ*g/mL) group at 24 h (^###^*p* < 0.001). § indicates significant LPS-mediated TNF*α* release compared to the untreated group (^§§§^*p* < 0.001).

As shown in Figure 6, the production of TNF*α* is only weakly stimulated by TiO_2_ NPs alone (Figure 6(a)), whereas, in LPS-primed macrophages, TNF*α* release was markedly increased (Figure 6(b)). Further, simultaneous exposure of macrophages to TiO_2_ NPs and LPS dose-dependently enhanced TNF*α* production (Figure 6(b)), which is well beyond the levels observed with TiO_2_ NPs or LPS prime alone. Notably, TiO_2_ NPs at low concentration (100*μ*g/mL) induced significantly more TNF*α* production than Ta NPs at high concentration (500 *μ*g/mL) in LPS-primed macrophages. This suggests that TiO_2_ NPs synergized with LPS to stimulate the production of TNF*α*. Whereas, Ta NPs did not exert such synergistic effect (Figure 6(b)).

Similar to TNF*α*, little IL-1*β* secretion was detected in macrophages challenged with Ta NPs alone up to 24 hours. TiO_2_ NPs (500 *μ*g/mL) stimulated a significantly higher IL-1*β* level than Ta NPs in unprimed macrophages, starting at 3h (Figure 6(c)). After LPS priming, much higher IL-1*β* levels were detected in TiO_2_-treated macrophages. TiO_2_ NPs further elevated IL-1*β* release in a time- and dose-dependent manner (Figure 6(d)), higher than LPS or TiO_2_ NPs alone. In contrast to the TNF*α* result, the Ta NP groups stimulated a negligible IL-1*β* increase after LPS priming (Figure 6(d)) compared to the untreated group. This indicated that the IL-1*β* level was not entirely dependent on LPS. In summary, these results suggest that LPS aggravates inflammation in macrophages. Also, TiO_2_ NPs synergized with the LPS effect to increase the production of TNF*α* and IL-1*β* while Ta NPs did not. Thus, TiO_2_ NPs possess a more substantial proinflammatory effect than Ta NPs.

### 3.7. Scavenging of ROS Attenuates TiO_2_-Induced Cell Death and Proinflammatory Cytokine Release

**Figure 7:**
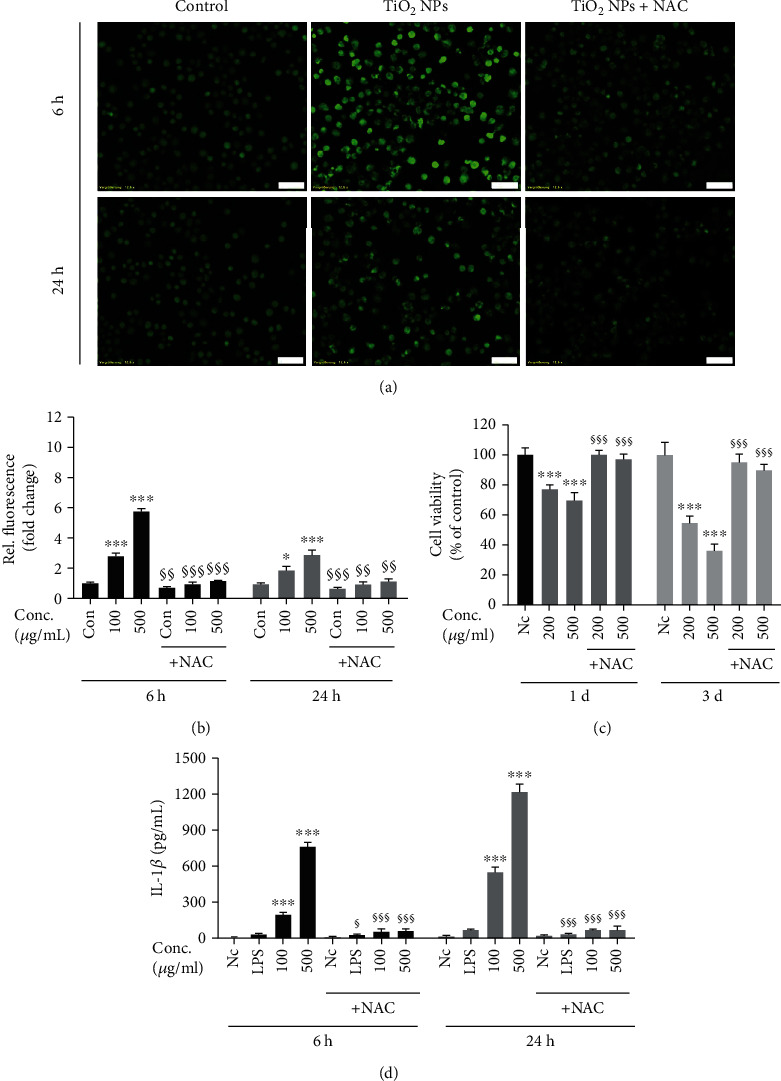
Protective role of ROS scavenger (NAC) on TiO_2_ NP-induced cell death and IL-1*β* release. LPS-primed macrophages were preincubated for 30min with/without NAC (5mM), and then cotreated with TiO_2_ NPs at the indicated doses for 6 and 24h. (a) Following exposure, cells were incubated with DCFH-DA (20*μ*M) for 30 min at 37°C, and ROS production was detected by fluorescence microscopy (20x). Scale bar: 50*μ*m. (b) Relative quantification of ROS generation (fold change). The protective role of NAC on (c) cell viability and release of (d) IL-1*β* were measured by CCK-8 and ELISA, respectively. Data are presented as mean + SD of three identical experiments performed in three replicates. ^∗^ indicates significant difference as compared to the control (^∗^*p* < 0.05, ^∗∗^*p* < 0.01, and ^∗∗∗^*p* < 0.001); § indicates significant inhibitory effect of NAC on cell death and proinflammatory cytokine generation (^§^*p* < 0.05, ^§§^*p* < 0.01, and ^§§§^*p* < 0.001).

Intracellular ROS are key effectors in signal transduction and are proposed to be associated with cell death and inflammation. ROS generation has been proven crucial for NP-induced NLRP3 inflammasome activation and subsequent IL-1*β* release [33, 34]. Therefore, we applied antioxidant NAC to prove the effect of ROS on TiO_2_ NP-induced cell death and IL-1*β* release. NAC is a potent thiol-containing antioxidant that can act as a precursor of glutathione (GSH) and can also directly scavenge free radicals (e.g., H_2_O_2_ and OH^−^) [35]. As shown in Figures 7(a) and 7(b), the fluorescence intensity was lower in NAC-treated cells in comparison to cells treated with TiO_2_ NPs alone at 6 and 24h, indicating that NAC successfully antagonized ROS accumulation elicited by TiO_2_ NPs in macrophages. Moreover, NAC treatment significantly mitigated cell death (Figure 6(c)) and potently blocked IL-1*β* release induced by TiO_2_ NPs (Figure 6(d)) after LPS prime. These results indicated that ROS inhibition by NAC could rescue TiO_2_ NP-induced nanotoxicity and mitigate IL-1*β* production in macrophages.

## 4. Discussion

Ta-based implants have been widely used in primary and revision TJA. Nevertheless, knowledge about the possible effect of their wear products on peri-implant cells remains limited to date. Therefore, understanding the local cellular responses to nanoscale orthopedic wear particles will provide a new area for comprehension of aseptic loosening and offers new scientifically based recommendations to better design suitable prosthetic interfaces and scaffolds. To the best of our knowledge, the present paper is the first *in vitro* analysis investigating Ta NPs and TiO_2_ metallic NPs side by side in terms of their biological responses on macrophages. We provide scientific evidence that Ta NPs are inert, nontoxic, and noninflammatory NPs *in vitro.* The results of this study offer novel evidence-based insights to further substantiate the clinical application of Ta-based implants [36, 37].

We found a slight but significant increase in macrophage viability in the presence of 20*μ*g/mL Ta NPs at 1 and 3 days. Similar results were found in the study by Wang et al. and Kang et al. showing that Ta NPs promote the proliferation of mouse MC3T3-E1 osteoblasts at low concentration (less than 20 *μ*g/mL) through the induction of autophagy [38, 39]. However, our CCK-8 and LDH results also demonstrated that Ta NPs, even when using higher concentrations (e.g., 50-500*μ*g/mL) and longer incubation times (up to 7 days), still resulted in high cell viability, which ultimately supports their good biocompatibility. In contrast, TiO_2_ NPs started to exhibit significant cytotoxicity at the 24-hour incubation time point. These aspects precisely show how important it is to fully characterize the cellular response of peri-implant cells to new implant materials and their resulting NPs. Therefore, the present study is of particular interest as it provides the first comparative analysis showing that Ta NPs induced less cytotoxicity in macrophages than NPs stemming from Ti implants.

Phagocytosis and endocytosis are responsible for the uptake and clearance of particles [27, 40]. However, to date, the mechanistic nature of TiO_2_ and Ta NP internalization in human macrophages remains obscure. Our study demonstrated that active Ta and TiO_2_ NP internalization by macrophages is mainly driven by phagocytosis. Further, TiO_2_ internalization is also mediated, to a lesser extent, by clathrin-dependent endocytosis and micropinocytosis. These results are supported by previous studies, which have shown that TiO_2_ particle internalization is mediated by phagocytosis and endocytosis in glial cells [28], H9c2 rat cardiomyoblasts [27], and rodent macrophages [41, 42]. The multiple uptake pathways of TiO_2_ NPs may partially explain why they were internalized more efficiently and why they induce more robust cytotoxicity, oxidative stress, and inflammatory cytokines compared to Ta NPs under the same conditions.

Increased bone resorption due to chronic inflammatory responses from wear particle-challenged macrophages underlies the pathogenesis of periprosthetic osteolysis [21]. TNF*α* is a master cytokine during inflammation and a potent inducer of other proinflammatory chemokines and cytokines. When focussing on inflammatory cytokines, the Ta-based surface seems to exert an anti-inflammatory effect compared to the Ti-based surface [43], suggesting that Ta substrates are more biologically inert and may provide a more favorable environment when applied as biomaterial. However, other studies reported that Ta-based surfaces could be more inflammatory than Ti-based [44] surfaces. This discrepancy may be attributed to the difference in surface elemental composition, modification methods, and culturing model (*in vitro*, *in vivo*, and *ex vivo*).

Our results demonstrated that Ta and TiO_2_ NPs induced slight TNF*α* production without LPS priming. After exposure to LPS, which mimicked the situation of a low-grade infection in addition to the presence of wear particles, TNF*α* secretion increased over time in both NP groups. This is mainly because LPS can stimulate TNF*α* production through binding to Toll-like receptor-4 and subsequent activation of transcription factor NF-*κ*B. It is important to note that the TNF level elevation over time in the LPS plus Ta NP group is insignificant compared to that of the LPS-only group. In contrast, TiO_2_ NPs act synergistically with LPS and further elevate TNF*α* production. This synergistic effect directly leads to a significantly higher amount of TNF*α* in the TiO_2_ NP group (100*μ*g/mL) compared to the Ta NP group (100 and 500*μ*g/mL), indicating that, in the form of nanoscale particles, Ta is less proinflammatory than Ti particles.

IL-1*β* is considered an essential proinflammatory mediator driving osteolysis at the bone-implant interface. Recent studies demonstrated that orthopedic wear particles mediate IL-1*β* release via activation of the NLRP3 inflammasome [45, 46], whose activation is a two-step process requiring both priming (e.g., bacterial LPS) and activation signals (e.g., nigericin, silica crystal, and wear particles). Supporting these data, dramatic elevation of IL-1*β* secretion was only observed after LPS priming and TiO_2_ NPs. This indicated that both LPS priming and wear particles are required to license NLRP3 inflammasome activation [47]. Infection (in our case simulated by LPS) thus could be a potent inducer of inflammation, dramatically amplifying wear particle-induced inflammation and, therefore, could be a risk factor for implant loosening. The current results are in line with previous data which showed that LPS contributes to the biological activity of wear particles by increasing the proinflammatory cytokine production in macrophages [46, 48]. It is important to note that 500 *μ*g/mL Ta NPs induced similar SSC elevation to that of 100 *μ*g/mL TiO_2_ NPs at 6 hours. However, Ta NPs stimulated almost no elevation of IL-1*β* in the supernatant over time, even after priming with LPS. This, again, supported the noninflammatory property of Ta NPs compared to TiO_2_ NPs. It also suggests that, in the context of peri-implant infection, Ta-based implants may help limit the peri-implant inflammation, or could be a proper choice in revision surgery due to periprosthetic joint infection (PJI).

Interestingly, TiO_2_ NPs alone induced a low but significant elevation of IL-1*β* over time. Given that all our particle samples were endotoxin-free, this effect may be due to other priming signals, such as TNF*α* [49] (Figure 6(a)). This fits the clinical picture in which increased values of IL-1*β* are detected together with TNF*α* in chronic low-grade peri-implant inflammation without any sign of infection.

Oxidative stress has been proposed to play a role in nanotoxicology and inflammatory reactions [31, 50]. Previous studies have revealed that, compared to Ti substrates, Ta substrates demonstrate lower ROS generation in osteoblasts and bone marrow stromal cells and, therefore, exhibit better cellular viability and osteoinductivity [23, 51]. In our study, we found that TiO_2_ NPs induced robust ROS elevation while Ta NPs induced negligible amounts of ROS. This distinct difference in ROS-generating potential could be mainly attributed to the difference in particle type. Nevertheless, we cannot exclude that there are other potential influential factors, e.g., uptake efficiency, size, morphology, and oxidation extent. Furthermore, TiO_2_ NPs demonstrated higher cytotoxicity and induced higher inflammatory cytokines than Ta NPs. Thus, it is feasible to speculate that the difference in cytotoxicity and IL-1*β* release is due to differences in NPs' oxidative potential. We further proved this notion using NAC, a general ROS scavenger. Our study demonstrated that scavenging ROS with NAC abrogated TiO_2_ NP-induced ROS production in macrophages. Furthermore, NAC mitigated TiO_2_ NP-induced cell death and abrogated IL-1*β* release. Similar results were found in previous studies in which ROS suppression by NAC protected cells from cell death [52, 53] and IL-1*β* release [54–57] in response to ROS-generating NPs. This further proved that ROS depression can mitigate NP-induced cell death and IL-1*β* production and, therefore, may alleviate peri-implant tissue and inflammation. Although NAC has been successfully applied in previous studies as a ROS scavenger, it has multiple effects on cells [58, 59], which complicates the interpretation of the results. More specific ROS inhibitors are needed in the future to identify the ROS source. Collectively, the differences in inducing oxidative stress could explain why Ta NPs are relatively inert opposed to promoting cell death and inflammation as previously described for TiO_2_, or other widely used NPs such as silica NPs [60]. Furthermore, targeting ROS may serve as a therapeutic way to mitigate wear particle-induced chronic inflammation and prosthetic loosening. However, the exact source of ROS, e.g., mitochondrial and NADPH oxidase, and their relative contribution to NP-induced inflammation, need further characterization.

## 5. Conclusions

For the first time in this study, we examined macrophages' cellular response to nanoscale Ta and Ti particles *in vitro*. We found that Ta, in the form of nanoscale particles, was “bioinert” and induced less cytotoxicity, ROS production, and inflammatory response compared to TiO_2_ NPs. Thus, when applied as TJA biomaterials, Ta-based implants may provide a more favorable peri-implant biological environment and less potential to contribute to aseptic loosening than Ti implants. Considering that multiple biomaterials have been applied in the TJA field, comparing Ta particles with additional other particle types (e.g., polyethylene, cobalt, and chromium) should be included in future studies to assess the value of switching from conventional implants to Ta-based implants.

## Data Availability

The data used to support the findings of this study are available from the corresponding authors upon request.

## Supplementary Materials

Supplementary Materials Supplementary Table 1: hydrodynamic radius measured by DLS. TiO_2_ and Ta nanoparticles (NPs) were dispersed in ddH_2_O, PBS, RPMI 1640, and RPMI 1640 containing 10% FBS at 250 *μ*g/mL. Hydrodynamic radius (nm) was determined by DLS, and data from major peaks were presented. Supplementary Figure 1: endotoxin contamination of TiO_2_ and Ta NPs. Quantitative evaluation of endotoxin contamination was determined by an LAL endotoxin assay kit. Data were representative of three independent experiments. The endotoxin level was calculated according to the standard curve. n.d.: not detectable. Supplementary Figure 2: spectral intensities of the Ta (a) and TiO_2_ (b) samples observed in the energy-dispersive X-ray (EDX) analysis system. The spectra were analyzed in high vacuum (accelerating voltage: 20kV; spot size: 5.5). No contamination of other elements were observed. Supplementary Figure 3: cytotoxic effects of TiO_2_ and Ta NPs on macrophages from 1 day to 7 days. (a, b) Cell viability was determined by CCK-8 assay at indicated time points. (c, d) Lactate dehydrogenase (LDH) leakage was evaluated by LDH assay after 24h. Viability and LDH release are normalized and expressed as mean + SD as percentage of untreated cells of three independent experiments (^∗^*p* < 0.05, ^∗∗^*p* < 0.01, and ^∗∗∗^*p* < 0.001). n.d.: not detectable. Nc: nontreated control. Supplementary Figure 4: cytotoxic effects of inhibitors on macrophages. Macrophages were incubated with different concentrations of uptake inhibitors, and cell viability was evaluated by CCK-8 assay. Viability is normalized and expressed as mean + SD as the percentage of untreated cells of three independent experiments. ^∗^*p* < 0.05, compared with control.
