# Supplementary material for: Investigation of Cytotoxicity, Oxidative Stress, and Inflammatory Responses of Tantalum Nanoparticles in THP-1-Derived Macrophages
Source: Mediators Inflamm. 2020 Dec 3;2020:3824593. doi: 10.1155/2020/3824593 (PMC7732397; doi:10.1155/2020/3824593)
Supplement: Supplementary Materials — Supplementary Table 1: hydrodynamic radius measured by DLS. TiO2 and Ta nanoparticles (NPs) were dispersed in ddH2O, PBS, RPMI 1640, and RPMI 1640 containing 10% FBS at 250 μg/mL. Hydrodynamic radius (nm) was determined by DLS, and data from major peaks were presented. Supplementary Figure 1: endotoxin contamination of TiO2 and Ta NPs. Quantitative evaluation of endotoxin contamination was determined by an LAL endotoxin assay kit. Data were representative of three independent experiments. The endotoxin level was calculated according to the standard curve. n.d.: not detectable. Supplementary Figure 2: spectral intensities of the Ta (a) and TiO2 (b) samples observed in the energy-dispersive X-ray (EDX) analysis system. The spectra were analyzed in high vacuum (accelerating voltage: 20kV; spot size: 5.5). No contamination of other elements were observed. Supplementary Figure 3: cytotoxic effects of TiO2 and Ta NPs on macrophages from 1 day to 7 days. (a, b) Cell viability was determined by CCK-8 assay at indicated time points. (c, d) Lactate dehydrogenase (LDH) leakage was evaluated by LDH assay after 24h. Viability and LDH release are normalized and expressed as mean + SD as percentage of untreated cells of three independent experiments (∗p < 0.05, ∗∗p < 0.01, and ∗∗∗p < 0.001). n.d.: not detectable. Nc: nontreated control. Supplementary Figure 4: cytotoxic effects of inhibitors on macrophages. Macrophages were incubated with different concentrations of uptake inhibitors, and cell viability was evaluated by CCK-8 assay. Viability is normalized and expressed as mean + SD as the percentage of untreated cells of three independent experiments. ∗p < 0.05, compared with control. [file 3824593.f1.zip › 3824593.f1/Supplementary Table 1 (1).pdf]

Supplementary Table 1.

Sample	Average SEM Size (nm)	Hydrodynamic radius (nm)			
		ddH_2O	PBS	RPMI	RPMI+10% FBS
Ta NPs	67.9±22.1 nm	67	186	111	112
TiO_2 NPs	54.3±14.6 nm	155	283	139	196
